# Phage Display Based Cloning of Proteins Interacting with the Cytoplasmic Tail of Membrane Immunoglobulins

**DOI:** 10.1080/1044667031000137584

**Published:** 2002-09

**Authors:** Roland Geisberger, Martin Prlic, Gertrude Achatz-Straussberger, Iris Oberndorfer, Elke Luger, Marinus Lamers, Reto Crameri, Ulrich Appenzeller, Jürgen Wienands, Michael Breitenbach, Fatima Ferreira, Gernot Achatz

**Affiliations:** 1 Department of Genetics and General Biology Hellbrunnerstraβe 34 Salzburg A-5020 Austria; 2 DRFZ Schumannstrasse 21/22 D-10117 Germany; 3 MPI for Immunobiology Stübeweg 51 Freiburg D-79108 Germany; 4 SIAF Obere Strasse 22 Davos CH-7270 Switzerland; 5 Department of Biochemistry I Universitaetsstrasse 25 Bielefeld D-33615 Germany

## Abstract

The reduced quantity and quality of serum immunoglobulins (sIgs) in mutant mice expressing truncated cytoplasmic tails of IgE and IgG1 indicate an active role for the cytoplasmic domains of mIgG1 and mIgE. We used phage display technology to identify candidate proteins able to interact with the cytoplasmic tail of mIgE. Using a murine cDNA B cell library displayed on the surface of phage as prey and the 28 amino acid long cytoplasmic tail of IgE as bait, we isolated phage encoding the murine hematopoietic progenitor kinase 1 (HPK1). Surface plasmon resonance analysis measurements confirmed affinity of HPK1 to the mIgE cytoplasmic tail and revealed association to other immunoglobulin isotypes as well. Immunoprecipitation experiments, using lysates from two B cell lines expressing nitrophenyl (NP) specific mIgE molecules showed co-precipitation of IgE and HPK1. The interaction of HPK1 with the cytoplasmic domains of membrane immunoglobulins indicate an active role of the tails as part of an isotype specific signal transduction, independent from the Igα/Igβ heterodimers, and may represent a missing link to upstream regulatory elements of HPK1 activation.


*Developmental Immunology*, September 2002, Vol. 9 (3), pp. 127–134

### Phage Display Based Cloning of Proteins Interacting with the Cytoplasmic Tail of Membrane Immunoglobulins*

ROLAND GEISBERGER[a], MARTIN PRLIC[a], GERTRUDE ACHATZ-STRAUSSBERGER[a], IRIS OBERNDORFER[a], ELKE LUGER[b], MARINUS LAMERS[c], RETO CRAMERI[d], ULRICH APPENZELLER[d], JÜRGEN WIENANDS[e], MICHAEL BREITENBACH[a], FATIMA FERREIRA[a] and GERNOT ACHATZ[a,†]

[a]*Department of Genetics and General Biology, Hellbrunnerstraße 34, A-5020 Salzburg, Austria;* [b]*DRFZ, Schumannstrasse 21/22, D-10117, Germany;* [c]*MPI for Immunobiology, Stübeweg 51, D-79108 Freiburg, Germany;* [d]*SIAF, Obere Strasse 22, CH-7270 Davos, Switzerland;* [e]*Department of Biochemistry I, Universitaetsstrasse 25, D-33615 Bielefeld, Germany.*

The reduced quantity and quality of serum immunoglobulins (sIgs) in mutant mice expressing truncated cytoplasmic tails of IgE and IgG1 indicate an active role for the cytoplasmic domains of mIgG1 and mIgE. We used phage display technology to identify candidate proteins able to interact with the cytoplasmic tail of mIgE. Using a murine cDNA B cell library displayed on the surface of phage as prey and the 28 amino acid long cytoplasmic tail of IgE as bait, we isolated phage encoding the murine hematopoietic progenitor kinase 1 (HPK1). Surface plasmon resonance analysis measurements confirmed affinity of HPK1 to the mIgE cytoplasmic tail and revealed association to other immunoglobulin isotypes as well. Immunoprecipitation experiments, using lysates from two B cell lines expressing nitrophenyl (NP) specific mIgE molecules showed co-precipitation of IgE and HPK1. The interaction of HPK1 with the cytoplasmic domains of membrane immunoglobulins indicate an active role of the tails as part of an isotype specific signal transduction, independent from the Igα/Igβ heterodimers, and may represent a missing link to upstream regulatory elements of HPK1 activation.

*Keywords*: Antigen receptor; Cytoplasmic tail; HPK1; Phage display; Surface plasmon resonance

### INTRODUCTION

The B cell antigen receptor (Vitetta *et al.*, 1980; Reth, 1992) plays a central role in almost all processes along the developmental pathway of B cells, i.e. the generation, maturation, survival and activation of B cells. B cell antigen receptors (BCR) exist as a complex of membrane immunoglobulins (mIgs) and at least two other transmembrane polypeptides, called Ig-α and Ig-β, which connect the antigen receptor to the tyrosine phosphorylation pathways in the cell. In this complex, mIgs serve as isotype specific antigen receptors on the surface of B lymphocytes. All isotypes of mIgs can form a complex with Ig-α and Ig-β (Venkitaraman *et al.*, 1991) indicating an involvement of all isotypes in the signal transduction pathway. The earliest detectable biochemical event that follows BCR-aggregation is increased activity of protein tyrosine kinases (PTKs) of the Syk- and Src-family, which phosphorylate distinct tyrosine residues within conserved sequences present on the cytoplasmic portions of Ig-α and Ig-β (Reth, 1992). Phosphorylation of Ig-α and Ig-β occurs on the tyrosine residues of immuno receptor tyrosine-based activation motif (ITAM) and on the non-ITAM tyrosine 204 of Ig-α (Engels *et al.*, 2001). Once phosphorylated, ITAMs provide binding sites for SRC homology 2 (SH2) domains of the Src and Syk PTKs and the adaptor protein SLP-65 (Engels *et al.*, 2001), thus initiating several signaling pathways, the most important of which are the PLC-γ, the Ras and the PI3-kinase pathways (Reth and Wienands, 1997; Kurosaki, 1999). Various external stimuli such as cytokine binding, interaction with other cells as well as stress factors including osmotic shock, heat or ionizing irradiation can influence the signaling cascade initiated by BCR engagement and they can contribute to differential alteration of the pattern of gene expression.

Obviously, between Ig subclasses, there should be no differences in the transduction of signals regulating the central features of the immune system (Nussenzweig, 1997) such as the control of allelic exclusion, early cellular transitions and in the function of the membrane-bound immunoglobulin as a receptor for antigen capture

*Presented at the Proceedings of the 4th Germinal Center Conference, Groningen, The Netherlands, June 2002.

†Corresponding author. Address: Institut für Genetik, Hellbrunnerstraße 34, A-5020 Salzburg, Austria. Tel.: +43-662-8044-5764. Fax: +43-662-8044-144. E-mail: gernot.achatz@sbg.ac.at

ISSN 1044-6672 print © 2002 Taylor & Francis Ltd
DOI: 10.1080/1044667031000137584

and presentation. However, it has been clearly shown (Achatz *et al.*, 1997; Kaisho *et al.*, 1997; Luger *et al.*, 2001) that the cytoplasmic tails of IgG1 and IgE influence the quantity and quality of the immune response. These experiments show that, apart from the signal transduction pathways via Ig-$\alpha$ and Ig-$\beta$ an isotype-specific signal transduction pathway, initiated at the cytoplasmic tails of the mIgs, exists.

In the present work, we describe the use of the phage display technology for the isolation of candidate proteins able to interact with the cytoplasmic tail of mIgE and we present the murine hematopoietic progenitor kinase 1 (HPK1) (Kiefer *et al.*, 1996), a 97 kDa serine/threonine kinase, as a candidate protein that could modify signaling events originating at the cytoplasmic tail of mIgE.

### MATERIALS AND METHODS

### Construction of the Phage Display B Cell Library

To allow cDNA cloning with EcoRI and XhoI as methylation sensitive restriction enzymes, pJuFo phagemid (Crameri and Suter, 1993) was re-modeled (Crameri *et al.*, 2002) to pGA110. Splenocytes of 6 BALB/c mice were isolated and enriched for B cells (anti B220 monoclonal Ab RA3-6B2) yielding $3 \times 10^8$ cells. B cells were lysed and mRNA was isolated using the MACS mRNA kit. The 1st and 2nd cDNA strands were synthesized using the Stratagene cDNA synthesis kit according to the manufacturer's instructions. For 1st strand cDNA synthesis 5 μg mRNA was primed with an oligo(dT) primer containing an internal XhoI restriction site (5′ GAGAGAGAGAGAGAGAGAGAACTAGTCT-CGAGTTTTTTTTTTTTTTTTTT 3′). After 2nd strand synthesis, EcoRI adaptors were ligated to the blunted cDNA. cDNA inserts were ligated to pGA110 as EcoRI–XhoI fragments. Phages were generated as described before (Crameri and Suter, 1993; Crameri *et al.*, 2002).

### Coupling of the IgE Tail and Phage Panning

The IgE tail was synthesized as HIS-tagged peptide (HHHHHHKVKWVFSTPMQDTPQTFQDYA-NILQTRA). One hundred microliters of 1 mg/ml peptide (solved in PBS with 10 μg/ml BSA) were added to Qiagen Ni-NTA (Hisorb-strips) wells. After incubation for 2 h at 37°C, the wells were washed twice with 100 μl PBS and residual sites were blocked with BSA (10 μg/ml). One hundred microliters of the phage library ($10^{11}$ phagemids) was added per coated Ni-NTA well and incubated for 2 h at 37°C. Five phage panning rounds were performed as described (Crameri *et al.*, 2002).

### Semiquantitative PCR Analysis

The relative amount of cHPK-specific clones present after the 1st, 3rd and 5th panning round was determined by PCR using an cHPK specific 3′-primer an a 5′-specific vector primer (cHPK1 3′-primer: 5′ CTAGTGCGTCTGTTAG-GAGTCTG 3′; pGA110 5′-primer: 5′ GCAAACCGAA-ATCGCGAACCTGC 3′). Equal numbers of phages (titer was determined before) after each panning round were used for re-infection of *Escherichia coli*, followed by subsequent plating on agar plates. Total colonies were washed off and phagemid DNA was extracted. PCR conditions were: 2 min 95°C primary denaturation; 20 cycles repeating 45 s 95°C, 45 s 59°C and 1 min 72°C; 10 min 72°C final extension.

### Surface Plasmon Resonance Analysis

For surface plasmon resonance analysis two different sensor chips were used. (A): IgE-tail peptide (1 mg/ml diluted in PBS) was coupled to Flowchannel one (Fc1) of sensor chip CM5 (BIACORE) according to manufacturer's instructions. 1683 response units (RUs) were coupled to Fc1. As reference, a comparable amount of a random peptide (1704 RUs) was coupled to channel two (Fc2). CM5 measurements were performed by injecting $10^9$ cfu (suspended in TBS) for 1 min. Elution was done for 100 s. Phagemids were removed from the surface by injecting 100 mM NaOH.

(B): The NTA sensor chip was pre-treated and coupled as described by the manufacturer. One hundred microliters of HIS-tagged peptide (150 nM in 0.01 M Hepes, 0.15 M NaCl, 50 μM EDTA and 0.005% surfactant P20) was injected. Measurements were performed by injecting phagemids (diluted to $10^9$ cfu in TBS) for 1 min followed by 100 s of elution. The NTA sensor chip was regenerated for 5 min, followed by attachment of the next peptide. Four hundred (±25) RUs were coupled for each HIS-tagged Ig-tail. Binding curves were calculated using the BIA-viewer software program.

Peptides:

IgG1: (6 × H) KVKWIFSSVVELKQTLVPEYKNMI-GQAP

IgG2a: (6 × H) KVKWIFSSVVELKQTISPDYRNMI-GQGA

IgG2b: (6 × H) KVKWIFSSVVELKQKISPDYRNMI-GQGA

IgG3: (6 × H) KVKWIFSSVVQVKQTAIPDYRNMI-GQGA

IgE: (6 × H) KVKWVFSTPMQDTPQTFQDYA-NILQTRA

IgEΔTFQD: (6 × H) KVKWVFSTPMQDTPQYA-NILQTRA

IgM: (6 × H) KVK

Random peptide: SPHRCRIHPRPSKTMPTSSRPGH-RCDASTH

### B Cell Lysis, Immunoprecipitation and Co-immunoprecipitation

JW813/4 cells were seeded at a concentration of $5 \times 10^4$ cells/ml culture. Aliquots for analysis were taken at days 5, 7, 10, 12, 14 and 17 of incubation. Plasmacytoma cells, $5 \times 10^7$ were harvested by centrifugation at 1200 rpm for 5 min and lysed on ice for 10 min in 1 ml of lysis buffer (50 mM Tris, 137 mM NaCl, 0.1–1% Digitonin, (pH = 7.6) containing 1 μg/ml aprotinin, 1 μg/ml leupeptin, 1 mM phenylmethylsulfonylfluorid (PMSF) and 1 mM natriumorthovanadate). Insoluble material was removed by centrifugation at 10,000*g* for 10 min at 4°C. The supernatant, containing solubilized proteins, was stored at −20°C until further analysis.

The B-cell lysate was divided into two aliquots of 0.5 ml each. One aliquot was incubated with 10 μl of NP-sepharose beads (Biosearch Technologies Inc.). The control aliquot was incubated with CL-2B sepharose beads. Both aliquots were incubated overnight at 4°C under constant gentle agitation. The immune complexes were centrifuged at 1000 rpm for 1 min. The supernatant was discarded and the sepharose beads were gently washed with 0.5 ml lysis buffer by inverting the tube five times. The last two steps were repeated twice. Samples were eluted from the beads by incubating with 50 μl of non-reducing 1 × SDS gel-loading buffer (6 mM Tris pH = 6.8 (with HCl), 2% SDS, 10% Glycerol, 0.1% Bromophenol blue) at room temperature for 30 min. The supernatant was loaded onto a SDS-polyacrylamide gel and transferred by electroblotting to an Immobilon-PVDF (polyvinylidine fluoride) membrane according to standard procedures. Narrow strips of the membrane were incubated with either AP-coupled rat anti mouse IgE (EM95-3), or rabbit anti HPK1 serum followed by incubation with a goat anti rabbit AP-coupled antibody (SIGMA).

### RESULTS

### Isolation of Phagemids Displaying Proteins that Interact with the Cytoplasmic Tail of MIgE

For the generation of phagemids, the phage display B cell library, constructed in pGA110 (Crameri *et al.*, 2002), was superinfected with helper phage VCS M13. To guarantee full accessibility of the cytoplasmic tail during the phage display screening, the IgE tail was synthesized as HIS-tagged peptide and immobilized to Ni-NTA wells. Selective enrichment of phagemids was done as described before (Crameri and Suter, 1993). After five panning rounds phage enrichment was determined as the ratio between applied and eluted phage. More than 1000-fold enrichment was observed. Thirty randomly chosen single transductants from the last round of panning (fifth panning round) were sequenced. One specific sequence appeared several times and was selected for further characterization. Database comparison identified this sequence as HPK1 (Kiefer *et al.*, 1996). Detailed analysis of the insert showed that the phage displayed 87 amino acids of the C-terminal part of HPK1 (cHPK1). To verify specific phage enrichment during the panning rounds we performed a semiquantitative PCR analysis (Fig. 1a), where we amplified the phagemid insert after the first, third and fifth panning round using an insert specific and a vector specific primer. A three-fold increase of cHPK1 insert between the first and the fifth panning round could be detected, indicating a specific interaction of cHPK1 protein with the cytoplasmic tail of mIgE. We further tested the binding capacity of phagemids displaying cHPK1 using surface plasmon resonance analysis (Fig. 1b). The HIS-tagged mIgE cytoplasmic tail and a random peptide as control were covalently coupled to a CM5 (carboxymethylated dextran matrix) sensor chip. Phagemids, displaying

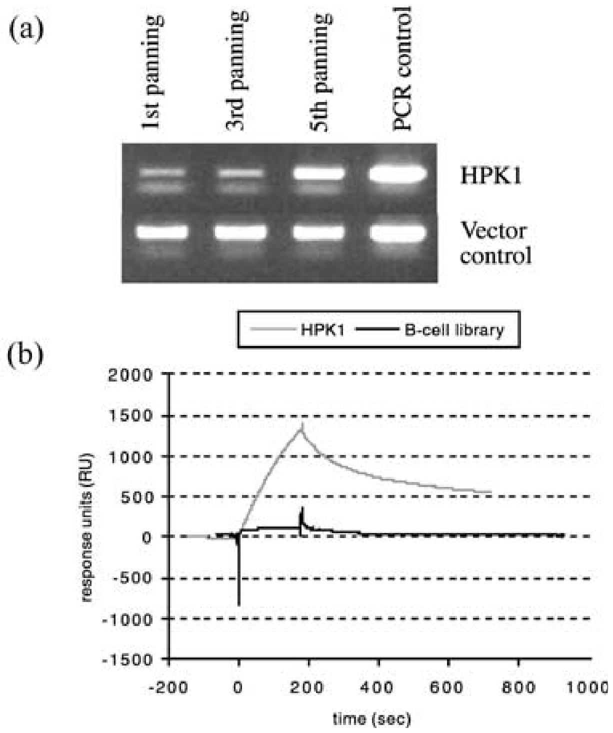

FIGURE 1 (a) The relative amount of cHPK-specific amplification product present after the 1st, 3rd and 5th panning round was determined by a semiquantitative PCR analysis using a cHPK specific 3′-primer and a 5′-specific vector primer. Selective enrichment of cHPK1 displaying phages, represented by a higher percentage of cHPK1 phagemids in the total phage population was observed, indicating specific interaction between cHPK1 protein and the cytoplasmic tail of IgE. (b) Specificity of the physical interaction between cHPK1-displaying phagemids and the cytoplasmic tail of IgE was investigated with surface plasmon resonance analysis, using a CM5 sensor chip with a covalently coupled IgE tail peptide. The complete phage display cDNA library served as control. The whole library showed a weak, but measurable affinity for the IgE tail, indicating the existence of phages displaying proteins with specificity for the IgE tail. In contrast, the cHPK1 displayed protein showed a remarkable increase of affinity for the cytoplasmic tail of IgE. cHPK1, $1 \times 10^9$, displaying phages were injected over a period of 200 s. After the elution step, a relative increase in affinity of 500 RUs could be measured.

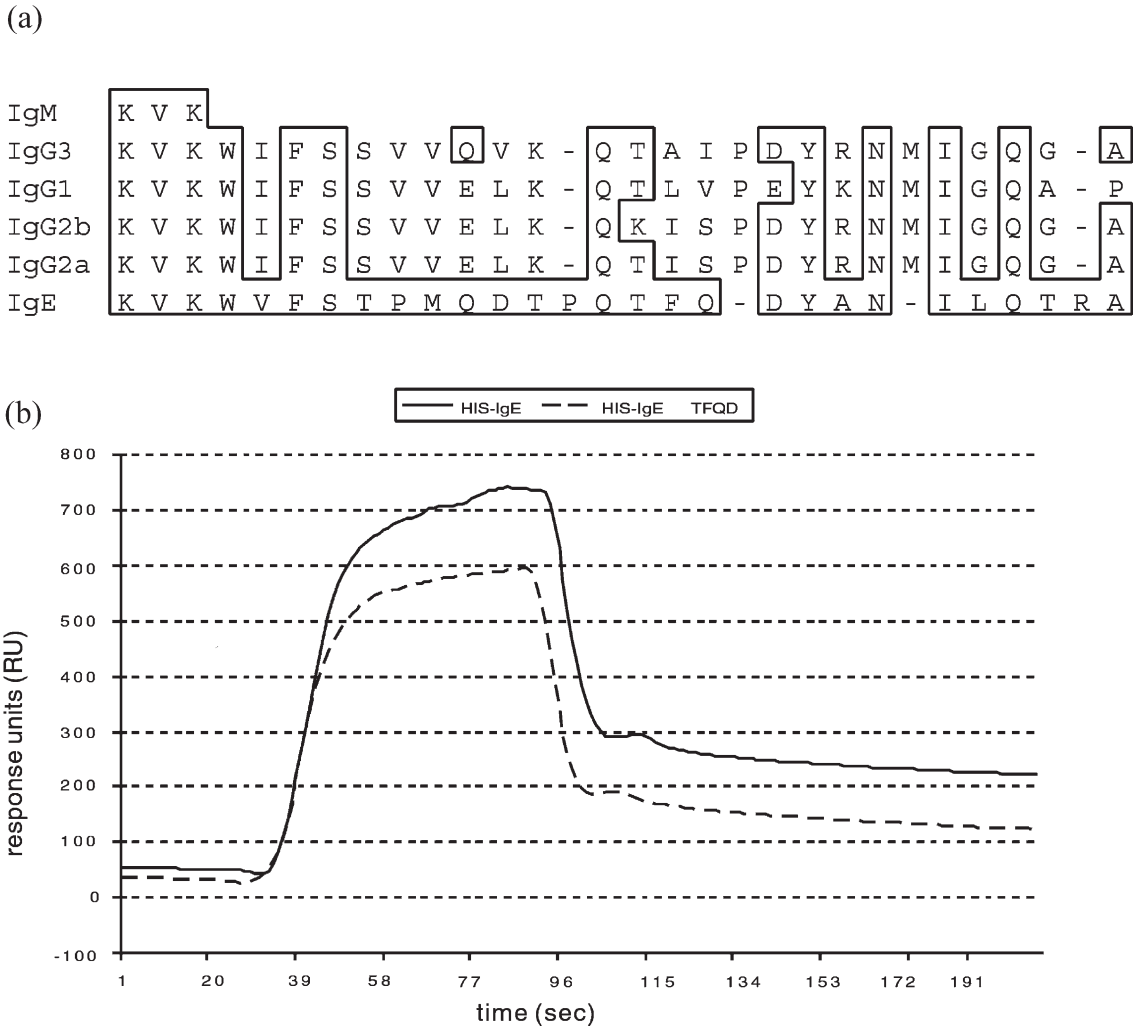

FIGURE 2 (a) Amino acid sequence alignment of the cytoplasmic tails of IgM, IgG3, IgG1, IgG2b, IgG2a and IgE. Amino acid residues that match IgE exactly are boxed. (b) To test whether the serine–threonine kinase binding motif T-F-Q-D influenced the binding of HPK1 to the cytoplasmic tail of IgE we immobilized a peptide lacking the TFQD amino acids (IgEΔTFQD) on a NTA-sensor chip. In comparison to the wild type IgE tail, the binding capacity of IgEΔTFQD is decreased by 47%, indicating a dramatic effect of the amino acid deletion on the binding affinity (Table I).

cHPK1 and the total B cell cDNA library were harvested, and titers were adjusted to $1 \times 10^{10}$ cfu/ml each. One hundred microliters of phagemid suspension ($1 \times 10^{9}$ cfu) was injected over a period of 200 s, followed by subsequent washing with TBS. The binding curves indicated a high relative affinity of cHPK1-carrying phagemids and a measurable affinity for the whole B-cell library.

### Binding of cHPK1 to Cytoplasmic Tails of other Isotypes

We next determined whether cHPK1 and cytoplasmic Ig-tails other than IgE could interact. HIS-tagged cytoplasmic tails of the IgM, IgG1, IgG2a, IgG2b, IgG3 and IgE were synthesized and subsequently coupled to a $Ni2^{+}$-NTA sensor chip via the HIS-tag. The cytoplasmic domains of

TABLE 1 The HIS-tagged cytoplasmic tails of IgE, IgG1, IgG2a, IgG2b, IgG3 and a modified IgE tail (IgEΔTFQD) were investigated for their capacity to bind the cHPK1 phage during surface plasmon resonance analysis

| | Injection after 60 s | Elution after 100 s | |
|---|---|---|---|
| Ig-Isotype | Relative RUs | Relative RUs | % |
| IgE | 691 | 177 | 100 |
| IgEΔTFQD | 563 | 94 | 53 |
| IgG1 | 819 | 128 | 72 |
| IgG2a | 284 | 68 | 38 |
| IgG2b | 276 | 72 | 41 |
| IgG3 | 744 | 120 | 67 |
| IgM | 617 | ≥0 | ≥0 |
| Random peptide | 310 | ≥0 | ≥0 |

The same amount of peptide (RUs) was covalently coupled to a CM5 sensor chip. Binding values (response units, RU) were taken 60 s after injection and 100 s after elution. IgE values were set to 100%.

mIgs are short and range from only three amino acid residues for mIgM and mIgD (lysine–valine–lysine–COOH in both cases) to 28 residues for the other mIg subclasses (Fig. 2a). The binding capacity of cHPK1 to the different Ig-tails was determined (Table I). Phage $10^9$ were incubated over a period of 60 s, followed by a 100-s elution step. Interestingly, cHPK1 binds to all Ig-tails tested but with much lower affinity compared to the cytoplasmic tail of IgE. Measuring the affinity of cHPK1 to the IgE tail, the relative response units (RUs) increased by 691 in the association phase. After 100 s of elution, 177 RUs remained as relative binding value. A lower affinity was measured when we investigated the interaction with the cytoplasmic tail of IgG1. Although binding increased by 819 RUs, the remaining value after elution decreased to 128 RUs. IgG2a and IgG2b differ in their cytoplasmic tails in only one amino acid. Accordingly, the binding behavior was similar for both isotypes. With both tails a decrease of binding affinity after the elution step of about 60% was seen. The cytoplasmic tail of IgG3 showed an increase of 744 RUs in the binding phase and decreased to 120 RUs after elution. As control, we measured a HIS-tagged IgM tail and a HIS-tag deficient random peptide for their affinity for cHPK1. As expected, the three amino acids of IgM (KVK) are not sufficient for any physical interaction between the two molecules. A comparable result was achieved with the HIS-tag deficient random peptide. By setting the measured relative RUs observed in the IgE measurement to 100%, we observed a 28–62% decrease of the relative binding affinity between the different Ig isotypes. Summarizing, BIACORE sensor chip technology clearly showed that cHPK1 is able to bind cytoplasmic tails other than IgE, but the relative affinity to the cytoplasmic tail of mIgE is higher.

Finally, we measured the binding affinity of the cHPK1 phage to a modified cytoplasmic tail of IgE where we deleted the four amino acids T-F-Q-D, a serine/threonine kinase binding motif (S/T-x-x-D/E) (Litchfield *et al.*, 1990) that could serve as a docking site for a serine/threonine kinase. BIACORE analysis (Fig. 2b; Table I) showed that deletion of TFQD in the cytoplasmic tail of IgE resulted in a remarkable decrease of affinity for cHPK1. After elution of the phage, only 53% of the affinity of the native IgE tail could be measured. We conclude that the four conserved amino acids are necessary for efficient binding of cHPK1 *in vitro* and may present a potent docking site for the serine/threonine kinase HPK1. A similar motif is also present in IgG1, IgG2b and IgG2a as amino acid sequence S-V-V-E. Only IgG3 lacks this consensus or uses a modified version in form of S-V-V-Q.

### HPK1 Co-precipitates with mIgE Expressing Plasmacytomas

The lymphoid myeloma lines J558L-Em and JW813/4 express mIgE molecules with specificity for the hapten 4-hydroxy-3-nitrophenylacetyl (NP). Both cell lines were stably transfected with a mIgE expressing genomic construct. However, after fresh splitting of the cell culture IgE surface expression can only be observed during a small time window. To find the best conditions for mIgE- and HPK1 expression in the JW813/4 cell line, cells were monitored over a period of 17 days using FACS analysis with FITC-coupled α-IgE (EM95-3) (Fig. 3a) antibodies and immunoblotting for detection of IgE and HPK1 (Fig. 3b).

For FACS analysis, $10^6$ cells were labeled with FITC-coupled α-IgE (EM95/3) antibodies and the number of cells expressing mIgE was measured (Fig. 3a). mIgE surface expression peaked at day 10. For immunoblotting aliquots of $2 \times 10^6$ cells were collected for each time point and lysed in 200 μl of lysis buffer. Ten microliters of each sample was separated by SDS-PAGE under non-denaturing conditions, transferred to polyvinyl membranes and probed for IgE and HPK1 presence (Fig. 3b). While HPK1 expression decreased during the time course, IgE expression reached a maximum at day 12, followed by a rapid decrease. Because membrane bound IgE is evidently the most crucial parameter for co-precipitation, we decided to continue experiments with cells from 10-day-old cultures. This time point is a compromise between surface expression of mIgE, and an acceptable level of HPK1 expression.

Immunoprecipitation and co-immunoprecipitation were performed with J558L-Em and JW813 cell lysates. We failed to co-precipitate mIgE with HPK1 when digitonin in a concentration of 1 or 0.5% was used. Best results were observed with a 0.1% digitonin concentration. In Fig. 3c, results of a co-precipitation experiment with JW813 cells under these conditions are depicted. Lysates of JW813 cells ($5 \times 10^7$ cells in 1 ml lysis buffer) were incubated with NP-Sepharose or control Sepharose. After precipitation complexes were separated on non-reducing polyacrylamide gels. Stripes of the membrane were then either stained with a rabbit anti-HPK1 antiserum followed by an alkaline phosphatase labeled anti-rabbit antibody to reveal HPK1 or with alkaline phosphatase labeled monoclonal anti-IgE antibodies. The results in Fig. 3c clearly show the co-precipitation of the unstimulated IgE antigen receptor complex and HPK1 protein.

### DISCUSSION

In the present work, we described the use of the phage display technology to isolate candidate proteins able to interact with the cytoplasmic tail of mIgE. Several theoretical objections could be raised against using this technology for finding interacting proteins. First, the pJuFo system is a prokaryotic system. Proteins that are presented on the surface of the phage may not be folded properly and therefore have a decreased specificity due to missing modifications. Second, the putative ligand could need phosphorylated residues in order to bind,

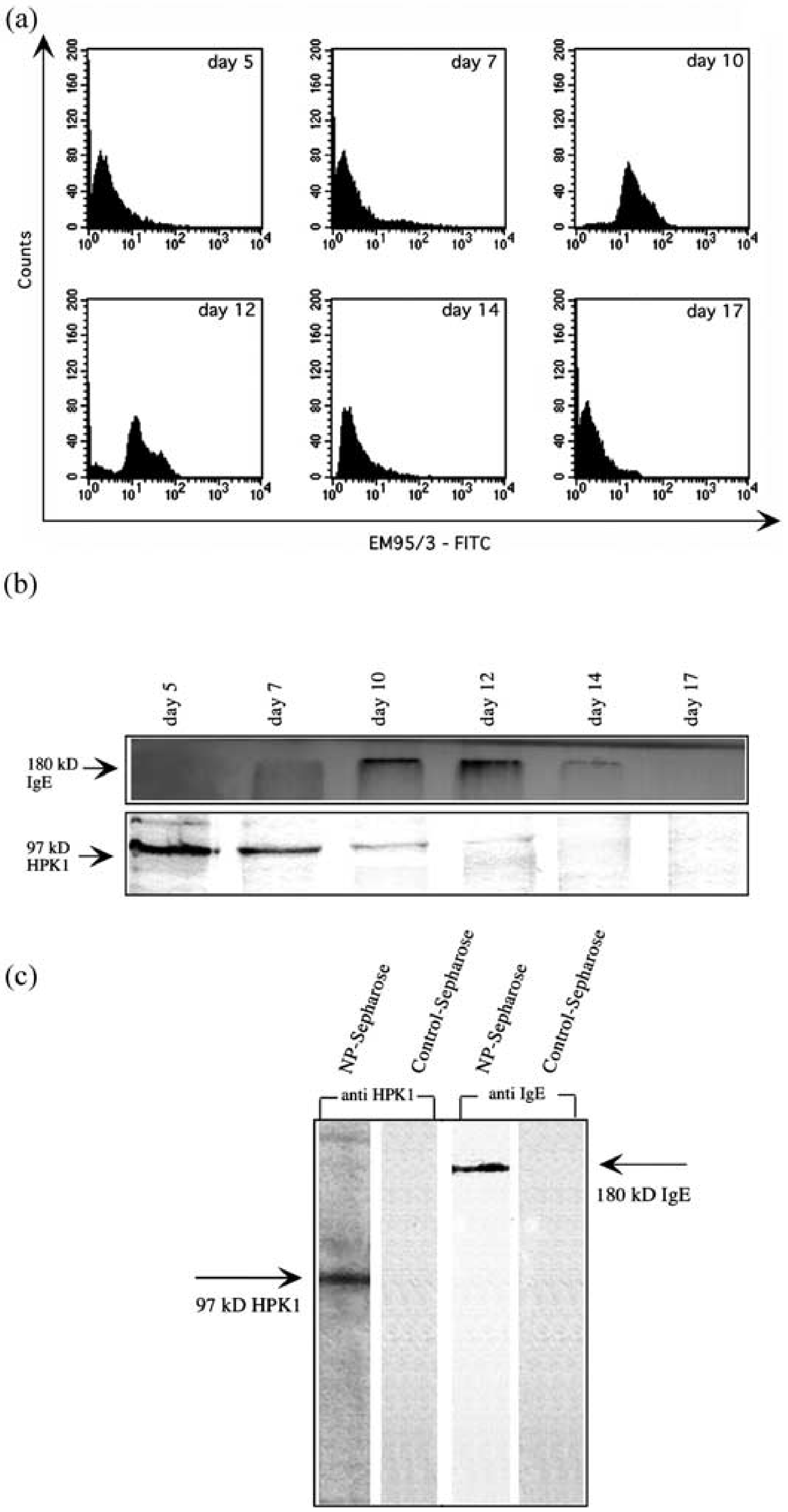

FIG. 3 (a) mIgE surface expression in the JW813/4 cell line was monitored over a period of 17 days. Flow cytometric analysis was performed with FITC-coupled α-IgE EM95-3 antibodies. Dead cells were excluded using propidium iodide. mIgE expression peaked at day 10. (b) Immunoblotting experiment for the detection of IgE and HPK1protein in the cell line. IgE expression nearly matched with the FACS experiment (a) and peaked at day 12, while HPK1 protein expression showed the maximum level at day 5. For the co-immunoprecipitation 10-days-old cells were used. (c) JW813 cells were lysed and precipitated with NP-sepharose and control sepharose. Both immune complexes were separated on a non-reducing polyacrylamide gel and transferred onto a PVDF-membrane and developed with AP-coupled rat anti mouse IgE (EM95-3), or rabbit anti HPK1 serum followed by incubation with a goat anti rabbit AP-coupled antibody. Both HPK1 and mIgE were detected in the same precipitate.

which was not provided by the synthetic IgE tail peptide. Third, the vicinity of the Igα/Igβ sheath could be crucial for the interaction. Finally, a potential binding partner might need the vicinity of the "dimeric" tail, as it naturally occurs in the cell, for efficient interaction. We point to this issue, because we failed to isolate candidate genes using the yeast-two-hybrid system. We reasoned that the phage display system could resolve this problem: HIS-tagged peptides, bound to NTA (nitrilo-triacetic-acid)-wells, theoretically can float freely in solution and thus present dimeric motifs to possible interacting partners during the selection procedure.

Indeed, we were able to isolate a potential candidate, the HPK1. The specific interaction of HPK1 with the cytoplasmic tail of IgE was demonstrated in different and independent experiments. Using surface plasmon resonance analysis, we could show that cHPK1 directly interacts with the cytoplasmic tail of IgE. However, with the exception of IgM, HPK1 interacted also with the cytoplasmic domains of other Ig isotypes, albeit with a lower affinity. This observation raises the possibility that isotype-dependent signal transduction pathways exist in parallel to the well-characterized signal transduction pathways via the Igα/Igβ sheath proteins.

The insert isolated from the phages encoded 87 amino acids that fully matched the carboxy-terminal part of the murine (HPK1) (Kiefer *et al.*, 1996), a 97-kDa serine/threonine kinase. A serine/threonine kinase binding motif (S/T-x-x-D/E) earlier identified by Litchfield *et al.* (1990) is present in the IgE tail (T-F-Q-D) and could serve as a docking site for HPK1. A similar motif is also present in IgG1, IgG2b and IgG2a as amino acid sequence S-V-V-E. Only IgG3 lacks this consensus or uses a modified version in form of S-V-V-Q. To show the importance of this binding site for the interaction with HPK1 we used a modified IgE tail, lacking the four amino acids and repeated the BIACORE affinity measurements. The affinity of HPK1 for the modified IgE tail was dramatically reduced; a 47% reduction of affinity was measured, indicating the importance of the T-F-Q-D motif in the binding of HPK1 to the tail of IgE.

Our results indicate that the interaction of HPK1 and the cytoplasmic tail of IgE are phosphorylation independent, and additionally independent of the vicinity of the Igα/Igβ sheath. We point to this issue because the recently identified B cell specific adaptor protein SLP 65 (BASH or BLNK) (Fu *et al.*, 1998; Goitsuka *et al.*, 1998; Wienands *et al.*, 1998) containing SH2 domains, was shown to interact physically with the HPK1 protein in a phosphorylation-dependent manner (Sauer *et al.*, 2001; Tsuji *et al.*, 2001). However, this interaction was only visible after stimulation of the surface antigen receptor with anti BCR specific antibodies. To mimic a situation which resembles the original cloning experiment and the BIACORE-measurements, our experiments were performed with unstimulated mIgE expressing cell lines.

HPK1 was shown to play a central role in the network of cytoplasmic signaling in mice. HPK1 is expressed in nearly all embryonic tissues, but becomes restricted to hematopoietic organs (bone marrow, spleen, thymus) in adult mice. HPK1 belongs to the family of germinal center kinases (GCK), which all feature a proline-rich C-terminal regulatory domain (Liou *et al.*, 2000) and a N-terminal kinase domain, which shows homology to the yeast pheromone pathway element STE20. Although the physiological relevance of HPK1 remains to be determined, several factors promoting HPK1 activation have already been identified. These activators include interleukin-1 (IL-1), tumor necrosis factor-α (TNF-α), transforming growth factor-β (TGF-β) (Zhou *et al.*, 1999), erythropoietin (Nagata *et al.*, 1999), epidermal growth factor (EGF) (Anafi *et al.*, 1997), cellular injury (heat, UV and ionizing irradiation, chemotherapeutic drugs) and osmotic shock (Kiefer *et al.*, 1996). Furthermore, *in vitro* assays revealed potent autophosphorylation of HPK1 (Kiefer *et al.*, 1996).

Upstream elements, which finally lead to HPK1 activation are still poorly understood. HPK1 is suggested to act immediately downstream of membrane proximal signaling elements such as transmembrane receptors or small G-proteins (Kiefer *et al.*, 1996). Binding to tyrosine kinases is suggested as well (Anafi *et al.*, 1997). Most likely, translocation to membrane elements is accomplished by adaptor proteins containing src-homolgy 2 and 3 domains (SH2, SH3), which exhibit the potential to interact with the proline rich motifs of the C-terminal portion of HPK1 (Anafi *et al.*, 1997; Oehrl *et al.*, 1998; Ensenat *et al.*, 1999). The C-terminal binding motifs also play a crucial role in the connection of HPK1 with downstream signaling elements, which are definitely better investigated than their upstream counterparts. Based on their homology to the yeast pheromone pathway kinase STE20, the GCKs may link receptor activation to the regulation of mitogen activated protein kinase (MAPK) modules. Indeed, HPK1 could be identified as potent upstream activator of the c-jun N-terminal kinase (JNK) pathway via the phosphorylation of the MAPK kinase kinase (MAPKKK) MLK-3 (Kiefer *et al.*, 1996). Because caspases regulate the JNK pathway (Chen *et al.*, 1999), HPK1 activation could also depend on caspase-mediated cleavage. Beyond c-jun activation, HPK1 was shown to be involved in another central immune specific signaling pathway, which leads to activation of nuclear factor kappa-B (NF-κB) (Hu *et al.*, 1999). While some of the MAPKKKs preferentially act on a single MAPK module, others may stimulate more than one pathway. Thus, the existence of a broad spectrum of possible substrates for HPK1 may not be excluded.

In the present paper, we indicate the existence of a signaling cascade facilitated by the binding of unphosphorylated HPK1 to the cytoplasmic tails of membrane-bound immunoglobulins. Possibly, this additionally modifies the signal in either quality or quantity, thereby modifying downstream signaling events and gene expression. We further propose that the physical interaction between the cytoplasmic tails of mIgs and HPK1 represents the missing link to upstream regulatory elements of HPK1.

### Acknowledgements

Animal experiments were conducted in accordance with guidelines provided by the Austrian law on experimentation with live animals. The work was supported by the Austrian Science Foundation (S8809-MED), the Austrian National Bank (OENB grant: 9546) and the Swiss National Science Foundation (grant 31-63381.00).

### References

Achatz, G., Nitschke, L. and Lamers, M.C. (1997) "Effect of transmembrane and cytoplasmic domains of IgE on the IgE response", *Science* **276**, 409–411.

Anafi, M., Kiefer, F., Gish, G.D., Mbamalu, G., Iscove, N.N. and Pawson, T. (1997) "SH2/SH3 adaptor proteins can link tyrosine kinases to a Ste20-related protein kinase, HPK1", *J. Biol. Chem.* **272**, 27804–27811.

Chen, Y.R., Meyer, C.F., Ahmed, B., Yao, Z. and Tan, T.H. (1999) "Caspase-mediated cleavage and functional changes of hematopoietic progenitor kinase 1 (HPK1)", *Oncogene* **18**, 7370–7377.

Crameri, R. and Suter, M. (1993) "Display of biologically active proteins on the surface of filamentous phages: a cDNA cloning system for selection of functional gene products linked to the genetic information responsible for their production", *Gene* **137**, 69–75.

Crameri, R., Achatz, G., Weichel, M. and Rhyner, C. (2002) "Direct selection of cDNAs by phage display", *Methods Mol. Biol.* **185**, 461–469.

Engels, N., Wollscheid, B. and Wienands, J. (2001) "Association of SLP-65/BLNK with the B cell antigen receptor through a non-ITAM tyrosine of Ig-alpha", *Eur. J. Immunol.* **31**, 2126–2134.

Ensenat, D., Yao, Z., Wang, X.S., Kori, R., Zhou, G., Lee, S.C. and Tan, T.H. (1999) "A novel src homology 3 domain-containing adaptor protein, HIP-55, that interacts with hematopoietic progenitor kinase 1", *J. Biol. Chem.* **274**, 33945–33950.

Fu, C., Turck, C.W., Kurosaki, T. and Chan, A.C. (1998) "BLNK: a central linker protein in B cell activation", *Immunity* **9**, 93–103.

Goitsuka, R., Fujimura, Y., Mamada, H., Umeda, A., Morimura, T., Uetsuka, K., Doi, K., Tsuji, S. and Kitamura, D. (1998) "BASH, a novel signaling molecule preferentially expressed in B cells of the bursa of Fabricius", *J. Immunol.* **161**, 5804–5808.

Hu, M.C., Wang, Y., Qiu, W.R., Mikhail, A., Meyer, C.F. and Tan, T.H. (1999) "Hematopoietic progenitor kinase-1 (HPK1) stress response signaling pathway activates IkappaB kinases (IKK-alpha/beta) and IKK-beta is a developmentally regulated protein kinase", *Oncogene* **18**, 5514–5524.

Kaisho, T., Schwenk, F. and Rajewsky, K. (1997) "The roles of gamma 1 heavy chain membrane expression and cytoplasmic tail in IgG1 responses", *Science* **276**, 412–415.

Kiefer, F., Tibbles, L.A., Anafi, M., Janssen, A., Zanke, B.W., Lassam, N., Pawson, T., Woodgett, J.R. and Iscove, N.N. (1996) "HPK1, a hematopoietic protein kinase activating the SAPK/JNK pathway", *EMBO J.* **15**, 7013–7025.

Kurosaki, T. (1999) "Genetic analysis of B cell antigen receptor signaling", *Annu. Rev. Immunol.* **17**, 555–592.

Liou, J., Kiefer, F., Dang, A., Hashimoto, A., Cobb, M.H., Kurosaki, T. and Weiss, A. (2000) "HPK1 is activated by lymphocyte antigen receptors and negatively regulates AP-1", *Immunity* **12**, 399–408.

Litchfield, D.W., Arendt, A., Lozeman, F.J., Krebs, E.G., Hargrave, P.A. and Palczewski, K. (1990) "Synthetic phosphopeptides are substrates for casein kinase II", *FEBS Lett.* **261**, 117–120.

Luger, E., Lamers, M., Achatz-Straussberger, G., Geisberger, R., Infuhr, D., Breitenbach, M., Crameri, R. and Achatz, G. (2001) "Somatic diversity of the immunoglobulin repertoire is controlled in an isotype-specific manner", *Eur. J. Immunol.* **31**, 2319–2330.

Nagata, Y., Kiefer, F., Watanabe, T. and Todokoro, K. (1999) "Activation of hematopoietic progenitor kinase-1 by erythropoietin", *Blood* **93**, 3347–3354.

Nussenzweig, M.C. (1997) "Immune responses: tails to teach a B cell", *Curr. Biol.* **7**, R355–R357.

Oehrl, W., Kardinal, C., Ruf, S., Adermann, K., Groffen, J., Feng, G.S., Blenis, J., Tan, T.H. and Feller, S.M. (1998) "The germinal center kinase (GCK)-related protein kinases HPK1 and KHS are candidates for highly selective signal transducers of Crk family adapter proteins", *Oncogene* **17**, 1893–1901.

Reth, M. (1992) "Antigen receptors on B lymphocytes", *Annu. Rev. Immunol.* **10**, 97–121.

Reth, M. and Wienands, J. (1997) "Initiation and processing of signals from the B cell antigen receptor", *Annu. Rev. Immunol.* **15**, 453–479.

Sauer, K., Liou, J., Singh, S.B., Yablonski, D., Weiss, A. and Perlmutter, R.M. (2001) "Hematopoietic progenitor kinase 1 associates physically and functionally with the adaptor proteins B cell linker protein and SLP-76 in lymphocytes", *J. Biol. Chem.* **276**, 45207–45216.

Tsuji, S., Okamoto, M., Yamada, K., Okamoto, N., Goitsuka, R., Arnold, R., Kiefer, F. and Kitamura, D. (2001) "B cell adaptor containing src homology 2 domain (bash) links b cell receptor signaling to the activation of hematopoietic progenitor kinase 1", *J. Exp. Med.* **194**, 529–540.

Venkitaraman, A.R., Williams, G.T., Dariavach, P. and Neuberger, M.S. (1991) "The B-cell antigen receptor of the five immunoglobulin classes", *Nature* **352**, 777–781.

Vitetta, E., Pure, E., Isakson, P., Buck, L. and Uhr, J. (1980) "The activation of murine B cells: the role of surface immunoglobulins", *Immunol. Rev.* **52**, 211–231.

Wienands, J., Schweikert, J., Wollscheid, B., Jumaa, H., Nielsen, P.J. and Reth, M. (1998) "SLP-65: a new signaling component in B lymphocytes which requires expression of the antigen receptor for phosphorylation", *J. Exp. Med.* **188**, 791–795.

Zhou, G., Lee, S.C., Yao, Z. and Tan, T.H. (1999) "Hematopoietic progenitor kinase 1 is a component of transforming growth factor beta-induced c-Jun N-terminal kinase signaling cascade", *J. Biol. Chem.* **274**, 13133–13138.

